# MicroRNA miR-124 Controls the Choice between Neuronal and Astrocyte Differentiation by Fine-tuning Ezh2 Expression*

**DOI:** 10.1074/jbc.M113.525493

**Published:** 2014-05-30

**Authors:** Wen Hao Neo, Karen Yap, Suet Hoay Lee, Liang Sheng Looi, Piyush Khandelia, Sheng Xiong Neo, Eugene V. Makeyev, I-hsin Su

**Affiliations:** From the ‡ Division of Molecular Genetics and Cell Biology, School of Biological Sciences, College of Science, Nanyang Technological University, 60 Nanyang Drive, Singapore 637551, Singapore and; the § Medical Research Council Centre for Developmental Neurobiology, King's College London, New Hunt's House, Guy's Hospital Campus, London SE1 1UL, United Kingdom

**Keywords:** Gene Expression, Gene Regulation, MicroRNA (miRNA), Neurodifferentiation, Polycomb

## Abstract

Polycomb group protein Ezh2 is a histone H3 Lys-27 histone methyltransferase orchestrating an extensive epigenetic regulatory program. Several nervous system-specific genes are known to be repressed by Ezh2 in stem cells and derepressed during neuronal differentiation. However, the molecular mechanisms underlying this regulation remain poorly understood. Here we show that Ezh2 levels are dampened during neuronal differentiation by brain-enriched microRNA miR-124. Expression of miR-124 in a neuroblastoma cells line was sufficient to up-regulate a significant fraction of nervous system-specific Ezh2 target genes. On the other hand, naturally elevated expression of miR-124 in embryonic carcinoma cells undergoing neuronal differentiation correlated with down-regulation of Ezh2 levels. Importantly, overexpression of Ezh2 mRNA with a 3′-untranslated region (3′-UTR) lacking a functional miR-124 binding site, but not with the wild-type Ezh2 3′-UTR, hampered neuronal and promoted astrocyte-specific differentiation in P19 and embryonic mouse neural stem cells. Overall, our results uncover a molecular mechanism that allows miR-124 to balance the choice between alternative differentiation possibilities through fine-tuning the expression of a critical epigenetic regulator.

## Teaser

**Background:** Molecular mechanisms underlying reduced expression of Ezh2 during neural differentiation are poorly understood.

**Results:** miR-124 directly down-regulates Ezh2 expression to promote neuronal differentiation.

**Conclusion:** Ezh2 is an important target of miR-124 in the context of neuronal differentiation.

**Significance:** Our report represents a significant advance in understanding the contribution of a microRNA/epigenetic regulatory circuitry to cell fate determination in the neural lineage.

## Introduction

Since their discovery in 1993, microRNAs (miRNAs),^2^ 19–25-nucleotide-long non-coding RNA molecules, have emerged as versatile regulators of developmental and physiological processes in a large fraction of eukaryotic organisms (1, 2). There are estimated to be over 1000 miRNAs in the human genome, and more than 50% of human genes are predicted to be miRNA targets (3, 4). Mature miRNAs regulate their cognate mRNAs as a part of an miRNA-induced silencing complex containing an Argonaute protein subunit (5). In plants, miRNAs often bind to fully complementary target sites typically located in the mRNA 3′-UTRs, which leads to gene repression through Argonaute-dependent mRNA “slicing” (6). On the other hand, animal miRNAs tend to be partially complementary to their target sequences, which affords regulation of target mRNAs through translational inhibition and slicer-independent destabilization (7, 8).

miRNAs are known to be crucial for neuronal differentiation, because conditional ablation of the endoribonuclease Dicer, an essential component of the microRNA maturation pathway, in neural stem cells or progenitors leads to dramatic defects in survival and differentiation of newborn neurons (9–11). One of the most abundant and perhaps best studied miRNAs in the brain is miR-124 (12–16). miR-124 is derived from three independent genes (*miR-124-1*, *miR-124-2*, and *miR-124-3*) contributing to the increased mature miR-124 levels during neuronal differentiation (4, 17, 18). Interestingly, however, knock-out of just the *miR-124-1* gene in the mouse resulted in visible reduction of mature miR-124 levels, defective neuronal survival, and axonal outgrowth as well as smaller brain size (19).

miR-124 may regulate hundreds and possibly thousands of distinct target genes (18, 20–23). Important examples include genes encoding the SCP1 subunit of the global repressor of NS-specific genes REST, transcription factors Sox9 and cAMP-response element-binding protein, Notch ligand Jagged1, and the BAF53a subunit of a chromatin remodeling complex (24–27). We have previously shown that miR-124 also targets mRNA of Ptbp1 (polypyrimidine tract-binding protein), a global regulator of pre-mRNA splicing (11). Ptbp1 is expressed at high levels in non-neuronal cells and neuronal precursors, where it suppresses the utilization of neuron-specific alternative exons. During neuronal differentiation, Ptbp1 expression is reduced by miR-124, which triggers a switch in alternative splicing patterns among a wide range of transcripts. Ptbp1 additionally controls the abundance of several neuron-specific mRNAs through nuclear and cytoplasmic RNA quality control mechanisms (11, 23, 28). Collectively, these studies demonstrate that miR-124 regulates several molecular pathways critical for proper progression of neuronal differentiation.

Neuron-specific genes are frequently modified by Ezh2-mediated H3K27 trimethylation (3meH3K27) in stem cells, whereas both the Ezh2 levels and the density of 3meH3K27 marks are down-regulated upon neuronal differentiation (29–31). Interestingly, overexpression of miR-124 in hepatocellular carcinoma cells, where it is normally present at negligibly low levels, has been shown to reduce Ezh2 expression (32). However, whether miR-124 contributes to down-regulation of Ezh2 expression during neurogenesis has not been investigated.

To this end, we first expressed miR-124 in mouse neuroblastoma Neuro2a (N2a) cells and showed that this treatment was sufficient to up-regulate a significant fraction of neuron-specific Ezh2 target genes. We further found that in P19 cells undergoing neuronal differentiation, the Ezh2 protein level was significantly reduced in an inverse correlation with increasing expression of mature miR-124. Importantly, miR-124-specific antisense inhibitor restored Ezh2 expression in differentiating P19 cells, whereas disruption of the putative miR-124 target site in exogenously expressed Ezh2 3′-UTR abolished the miR-124-mediated down-regulation and led to reduced neuronal differentiation. A similar effect of miR-124-regulated Ezh2 expression on neurogenesis was also observed in differentiating embryonic mouse neural stem cells. Thus, our results implicate Ezh2 as an important miR-124 target in the context of neuronal differentiation.

## EXPERIMENTAL PROCEDURES

### 

#### 

##### Plasmids

To generate the EGFP reporter construct for miRNA screening, 3′-UTR of Ezh2 was PCR-amplified from RP24–191K13 BAC clone and subcloned into the NotI site of pEGFP-N1 vector (Clontech). miRNA expression vectors were modified from pEM157 vector (11). A ∼500-bp DNA fragment flanking precursor miRNA sequence of interest was PCR-amplified from human genomic DNA and subcloned into the SpeI and NotI site of the intronic region of dsRed2 in pEM157 vector. Various Ezh2 donor plasmids were modified from pRD-RIPE plasmid (33) by replacing EGFP with Ezh2 or Ezh2-3′-UTR at AgeI and BglII sites. The QuikChange site-directed mutagenesis kit (Stratagene) was used to destroy the miR-124 target site in Ezh2 3′-UTR (32).

##### Cells

HEK293T cells were cultured in DMEM/high glucose (PAA Laboratories, GmbH) supplemented with 10% fetal bovine serum (FBS), 1 mm sodium pyruvate, 2 mm l-glutamine, 100 units/ml penicillin, 100 μg/ml streptomycin, and 55 μm 2-mercaptoethanol (all from Invitrogen). P19 cells were routinely propagated in α-minimal essential medium (HyClone) supplemented with 2.5% FBS, 7.5% bovine calf serum, 100 units/ml penicillin, and 100 μg/ml streptomycin.

##### P19 Stable Cell Line

P19 stable cell lines were generated as described (33). For stable cell line selection, 2 μg/ml puromycin was added to the medium for 5 days. To turn on the Tet-inducible expression, doxycycline (Clontech) was added to a final concentration of 2 μg/ml.

##### Neuronal and Astrocyte Differentiation of P19 Cells

To differentiate P19 cells into neuron and astrocyte, we adapted a protocol as described before (34). Briefly, 1 × 10^5^ cells/ml P19 cells were allowed to aggregate in a bacterial grade Petri dish (Fisher) and treated with 1 μm all-*trans*-retinoic acid (Sigma). The medium was changed at day 2. Cell aggregates were dissociated into a single cell suspension with 0.25% trypsin-EDTA (Invitrogen) at day 4. Cells were seeded onto a tissue culture grade Petri dish (Corning Inc.) at 1 × 10^5^ cells/ml in neurobasal medium with N2 supplement (Invitrogen) and 0.4 mm l-glutamine (Invitrogen). To obtain neuron and astrocyte, cells were further cultured until day 6.5 and day 12, respectively, for immunofluorescence staining. For protein and RNA assays, cells were cultured until day 10 or 12, and samples were collected at the indicated time points.

##### Cell Culture, Nucleofection, and Differentiation of Neural Stem Cells (NSCs)

NSCs were obtained from cortices of mouse embryonic day 14 and maintained in the form of neurospheres in complete NeuroCult® NSC proliferation medium supplemented with 20 ng/ml recombinant human epidermal growth factor (rhEGF, StemCell Technologies). To overexpress EGFP, Ezh2 with artificial 3′-UTR (Ezh2), or Ezh2 with wild-type Ezh2 3′-UTR (Ezh2 WT 3′-UTR) in NSCs, 1.6 × 10^6^ cells were co-nucleofected with 0.5 μg of pmaxGFP (Amaxa) together with 2 μg of the respective expression construct and plated onto poly-d-lysine-coated wells. 24 h postplating, the medium was changed to neurobasal medium containing N2 supplement for differentiation. Upon 72 and 120 h of differentiation, cells were harvested for RNA isolation and analyzed using quantitative RT-PCR.

##### Semiquantitative and Quantitative RT-PCR Analysis

Total RNA was purified using TRIzol (Invitrogen) as recommended. Reverse transcription (RT) was performed using SuperScript III (Invitrogen) with random hexamer. cDNA was amplified with specific primers for *Ezh2*, *Suz12*, *Eed*, and *Hprt*. The abundance of transcripts of the housekeeping gene *Hprt* was used as a loading control. Quantification of PCR product was done using image processing software, ImageJ (National Institutes of Health). The primer sequences were as follows: mEzh2 forward, 5′-AACACCAAACAGTGTCCATGCTAC-3′; mEzh2 reverse, 5′-CTAAGGCAGCTGTTTCAGAGAGAA-3′; mEeD forward, 5′-CAACACCAGCCACCCTCTAT-3′; mEeD reverse, 5′-GAGAAGGTTTGGGTCTCGTG-3′; mSuz12 forward, 5′-AAACGAAATCGTGAGGATGG-3′; mSuz12 reverse, 5′-CCATTTCCTGCATGGCTACT-3′; mHprt forward, 5′-GCTGGTGAAAAGGACCTCT-3′; mHprt reverse, 5′-CACAGGACTAGAACACCTGC-3′. For quantitative RT-PCR (RT-qPCR), cDNA was amplified with specific primers using an ABI StepOnePlus real-time PCR system (Applied Biosystems) and KAPA SYBR Fast ABI Prism 2x qPCR master mix (KAPA Biosystems). Data were normalized to the expression levels of the *Hprt* mRNA. The primer sequences were as follows: mEzh2 forward, 5′-TCCATGCAACACCCAACACAT-3′; mEzh2 reverse, 5′-GGGTCTGCTACTGTTATTCGGAA-3′; mL1cam forward, 5′-GGACAGCTTGAGGGTAGTAGA-3′; mL1cam reverse, 5′-CTGAAGACCACAACTCTCCCA-3′; mSyp forward, 5′-CGGCACATAGGCATCTCCT-3′; mSyp reverse, 5′-GAGAGAACAACAAAGGGCCAA-3′; mGfap forward, 5′-ACCGCATCACCATTCCTGTAC-3′; mGfap reverse, 5′-TGGCCTTCTGACACGGATT-3′; mS100b forward, 5′-TGGTTGCCCTCATTGATGTCT-3′; mS100b reverse, 5′-CCCATCCCCATCTTCGTCC-3′; mHprt forward, 5′-CCAGACAAGTTTGTTGTTGGA-3′; mHprt reverse, 5′-TTTACTAGGCAGATGGCCACA-3′; mAscl1 forward, 5′-GCAACCGGGTCAAGTTGGT-3′; mAscl1 reverse, 5′-GTCGTTGGAGTAGTTGGGGG-3′; mAtf3 forward, 5′-GAGGATTTTGCTAACCTGACACC-3′; mAtf3 reverse, 5′-TTGACGGTAACTGACTCCAGC-3′; mDusp8 forward, 5′-TGACCCAAAACGGAATAAGC-3′; mDusp8 reverse, 5′-CCTGTATGCGTCGTCAGAAG-3′; mEn2 forward, 5′-GCTGGCACTACCGAAGGAG-3′; mEn2 reverse, 5′-ACCGTGAAGTGATAGCGTCTT-3′; mSypl2 forward, 5′-CGCACCTCGGACAAGTCTC-3′; mSypl2 reverse, 5′-CCCGAAGGCGAAAATAGCAAA-3′; mTpm1 forward, 5′-GGGCTGAGTTTGCAGAGAGA-3′; mTpm1 reverse, 5′-TCAGCTGGAGAGCAGACAGA-3′.

##### Site-directed Mutagenesis

The predicted miR-124 binding site was mutated by base pair changes using DpnI-mediated site-directed mutagenesis (Stratagene). The primer sequences were as follows (32): miR124-Del forward, 5′-GTTTTAAAATCAACTTTTTATCTCACCAGCTGCAAAGTGTTTTG-3′; miR124-Del reverse, 5′-CAAAACACTTTGCAGCTGGTGAGATAAAAAGTTGATTTTAAAAC-3′; miR124-Sub forward, 5′-CAACTTTTTATACGGAACTCACCAGCTGCAAAGTGTTTTG-3′; miR124-Sub reverse, 5′-CAAAACACTTTGCAGCTGGTGAGTTCCGTATAAAAAGTTG-3′.

##### miRNA Northern Blot Analysis

Five μg of total RNA samples were separated on a 15% denaturing polyacrylamide gel containing 8 m urea and 1× TBE. They were electrotransferred to Hybond N^+^ membrane (Amersham Biosciences) in 0.5× TBE at 2.5 mA/cm^2^ for 30 min. RNA was cross-linked to the membrane by UV irradiation (0.15 J/cm^2^), and the membrane was blocked with 6× SSC, 7% SDS at 42 °C for overnight. Hybridization probes were prepared by labeling the appropriate oligodeoxyribonucleotides using T4 polynucleotide kinase (New England Biolabs) and [γ-^32^P]ATP (PerkinElmer Life Sciences). The ^32^P-labeled probes were purified using Sephadex G-25 microspin columns (Geneaid) and added to the blocking solution. The hybridization was carried out for overnight at 42 °C. The membranes were washed four times with 3× SSC, 0.1% SDS at 42 °C and exposed to phosphorimaging plates.

##### Immunoblotting

Whole cell lysates were prepared by resuspending cells in Nonidet P-40 buffer (50 mm Tris-HCl, pH 8.0, 500 mm NaCl, 1 mm EDTA, 1% Nonidet P-40, 10% glycerol) and sonicated (Bioruptor). Protein concentration was determined using the Bio-Rad DC protein assay (Bio-Rad) and analyzed by a Tecan infinite F200 plate reader (Tecan). Protein samples were subjected to SDS-PAGE. Antibodies used in the study were anti-Ezh2 (Cell Signaling Technology), anti-Tuj1 (Covance), anti-Erk1/2 (Sigma), anti-Suz12 (Abcam), and anti-Eed (Millipore).

##### Immunofluorescence

Immunofluorescence staining was performed according to a standard protocol. Anti-Tuj1 antibody was purchased from Covance. Anti-Gfap (ab4674) antibody was purchased from Abcam. Slides were mounted with mounting medium (Ibidi, GmbH) containing DAPI (Invitrogen).

##### Locked Nucleic Acid Antisense Oligonucleotide Transfection

Differentiated P19 cells (day 6) were seeded in 6-well plates at 3 × 10^5^ cells/well in 2 ml of antibiotic-free medium and transfected with 1, 5, or 30 pmol/ml miR-124 locked nucleic acid antisense oligonucleotide (Exiqon) using Lipofectamine 2000 (Invitrogen). Total protein and RNA were isolated 96 h post-transfection.

##### Microarray Analyses

N2a cell pools expressing different Argonaute paralog blends were described previously (35). Of these, N2a-WT cells containing the EGFP(shLuc) transgene had a native Argonaute expression dominated by Ago2, N2a-A1 cells containing the Ago1(shAgo2-3′-UTR) transgene expressed predominantly Ago1, and N2a-A2 cells containing the Ago2(shAgo2-3′UTR) transgene overexpressed Ago2 (35). All three cell pools were cultured in DMEM (HyClone) containing 10% FBS (HyClone, characterized grade), 1 mm sodium pyruvate (Invitrogen), 100 IU/ml penicillin, 100 mg/ml streptomycin (Invitrogen), 5 μg/ml puromycin, and 2 μg/ml doxycycline (antibiotic-containing complete DMEM) for 72 h prior to siRNA/miRNA duplex transfections. One million cells were seeded per well of a 6-well plate in 2 ml of antibiotic-free complete DMEM and allowed to adhere for 1 h. 100 pmol of corresponding siRNA/miRNA duplex mixed with Lipofectamine 2000 and OptiMEM I (Invitrogen) was then added per well as recommended. Medium was changed 5 h post-transfection to antibiotic-containing complete DMEM, and the incubation was continued for another 24 h. Total RNAs were extracted with TRIzol (Invitrogen) and cleaned up using an RNeasy kit (Qiagen). The RNAs were hybridized with Agilent Mouse SurePrint G3 8 × 60K Gene Expression Microarrays as recommended. The data sets were normalized using RobiNA (36), and genes showing consistent miR-124-induced up- or down-regulation were shortlisted in Excel using appropriate -fold change and *t* test *p* value cut-offs.

## RESULTS

### 

#### 

##### Overexpression of miR-124 in Neuroblastoma Cells Up-regulates Neuron-specific Ezh2 Target Genes

**FIGURE 1.**
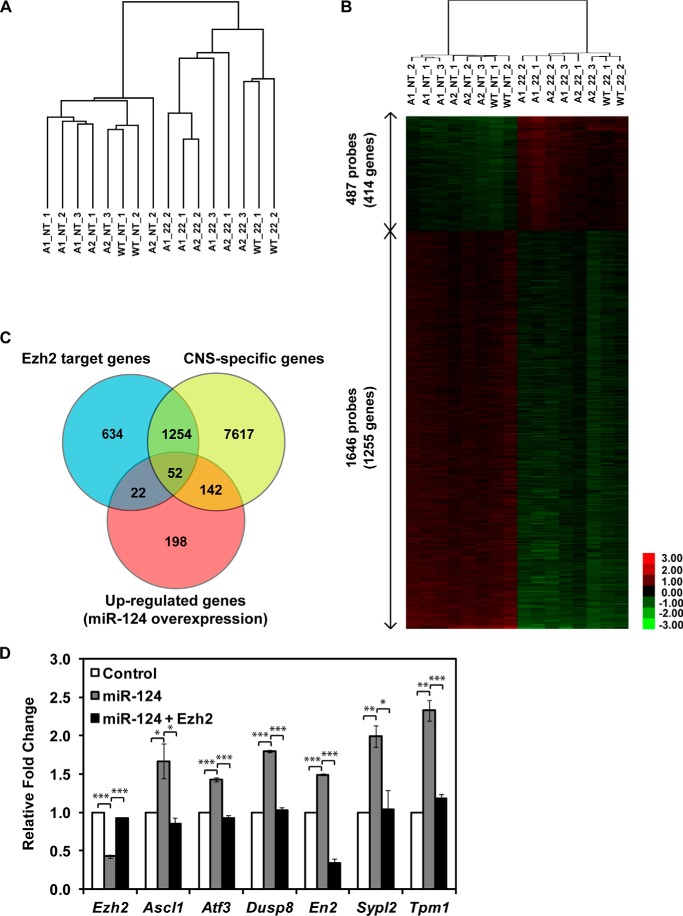
**miR-124 up-regulates neuron-specific Ezh2 target genes.** *A*, hierarchical clustering analysis of gene expression microarray data sets. miR-124-overexpressing N2a cell lines generated a distinctive gene expression pattern compared with non-transfected controls. Hierarchical clustering analysis (uncentered Pearson's correlation) of 16 samples and 24,288 genes separated untransfected samples from miR-124-transfected ones. *A1*, Argonaute-2 kinase-dead/Argonaute-1 reconstituted; *A2*, Argonaute-2 kinase-dead/Argonaute-2 reconstituted; *WT*, wild type; *NT*, non-transfected; *22*, 22-mer miR-124-transfected. *B*, a heat map was generated by performing unsupervised hierarchical clustering of 1669 differentially expressed genes with a *p* value cut-off of 0.001 and a -fold change (*FC*) range of 1.5 ≤ *FC* ≤ 0.67. Expression values for each gene in an individual array were calculated as log_2_ of the -fold change relative to the mean expression value in each array, which is represented as 0-fold in the *black*. The intensity of induction or repression is signified by the saturation of *red* or *green*, respectively. *C*, a significant fraction of miR-124-up-regulated genes are direct target genes of Ezh2. The Venn diagram shows that 414 genes are up-regulated (*FC* ≥1.5 and *p* < 0.001) by miR-124 expression, 194 of which are CNS-specific genes, and 52 of them are Ezh2 target genes that are significantly enriched in the miR-124-up-regulated CNS-specific gene list, as determined by Fisher's exact test (*p* = 7.03 × 10^−5^). *D*, selected genes from the miR-124-up-regulated CNS-specific gene list were validated by RT-qPCR. Their expression is further down-regulated by simultaneous expression of miR-124-resistant Ezh2. Significant differences between the indicated pairs were determined by two-tailed Student's *t* test with equal variance (*, *p* < 0.05; **, *p* < 0.01; ***, *p* < 0.001). A representative result with experimental triplicates from three independent experiments is shown. Data shown are mean (±) S.D. of triplicates.

To better understand miR-124 functions in the neural lineage, we transfected three N2a cell populations expressing distinct blends of Argonaute paralogs (N2a-WT, N2a-Ago1, and N2a-Ago2; see Ref. 35 for details) with either a synthetic siRNA-like duplex designed to deliver mature 22-mer miR-124 or a non-targeting siRNA control and analyzed the samples by Agilent gene expression microarrays. N2a cells were chosen because they express endogenous miR-124 at negligibly low levels (11, 35). Hierarchical clustering of the microarray data suggested that all three cell populations responded to miR-124 in a largely similar manner (Fig. 1*A*). This allowed us to pool individual population-specific data sets and focus on highly reproducible gene expression changes. In line with previous studies suggesting that this miRNA may directly regulate hundreds distinct mRNA targets, miR-124 consistently down-regulated 1255 genes (≥1.5-fold, *p* < 0.001; *t* test). Analysis of this subset by gene set enrichment analysis (37, 38) showed a dramatic overrepresentation of predicted miR-124 targets (*p* = 0; data not shown).

Interestingly, 414 genes were consistently up-regulated (≥1.5-fold, *p* < 0.001; *t* test) in miR-124-transfected N2a cells, presumably as a result of indirect effects (Fig. 1*B*). Strikingly, gene set enrichment analysis of this group uncovered a highly significant enrichment of genes previously identified as targets of 3meH3K27 histone modification or Suz12 either by ChIP-on-chip or ChIP-sequencing (data not shown), indicative of possible regulation of these genes by the PRC2 complex containing Ezh2 as a catalytic histone methyltransferase subunit. Notably, when we compared the list of the miR-124-up-regulated central nervous system (CNS)-specific genes (39) with genes known to be regulated by Ezh2 in stem cells (40), a highly significant overlap was detected (*p* = 7.03 × 10^−5^; Fisher's exact test) (Fig. 1*C*). On the other hand, the overlap between the corresponding subsets of miR-124-down-regulated CNS-specific genes and Ezh2 target genes could be explained by random sampling (*p* = 0.56; Fisher's exact test) (data not shown). Six of the miR-124-up-regulated Ezh2 target genes were further validated by RT-qPCR, and their expression levels were down-regulated by simultaneous expression of miR-124-resistant Ezh2 (Fig. 1*D*). These results suggested that miR-124 might regulate extensive subsets of genes by targeting Ezh2.

##### miR-124 Is a Potent miRNA Regulator of Ezh2 Expression

**FIGURE 2.**
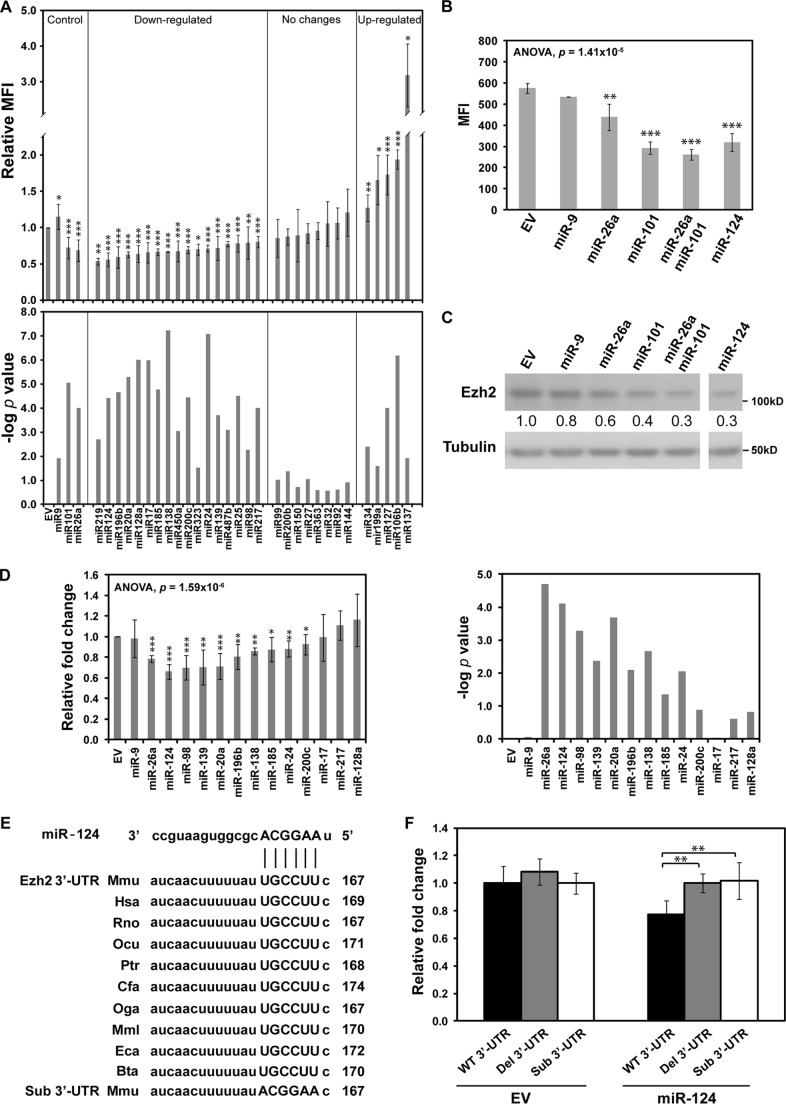
**miR-124 regulates Ezh2 expression by targeting Ezh2 3′-UTR.** *A*, screening for microRNAs regulating Ezh2 expression. An EGFP driven by the CMV promoter was fused to Ezh2 3′-UTR and used as a reporter in our fluorescence-based screening system. Individual miRNAs were expressed by dsRed2 containing vector pEM157. Both reporter and miRNA expression vectors were transfected into HEK293T cells and analyzed by FACS. miR-26a and miR-101 expression constructs were used as positive controls, and miR-9 was used as a negative control. EGFP mean fluorescence intensity (*MFI*) of the EGFP^+^ dsRed2^+^ population was assayed 48 h post-transfection. Relative mean fluorescence intensity was calculated after normalization against that of the empty vector (*EV*) control. *B*, miR-124 targets Ezh2 3′-UTR. Indicated miRNA expression vectors were co-transfected with EGFP reporter constructs in HEK293T cells. *C*, endogenous Ezh2 expression was analyzed by Western blot, and Tubulin was used as a loading control. The -fold change of Ezh2 was normalized to EV control and is shown *below* the Ezh2 blot. Data shown are representative of three independent experiments. *D*, the indicated miRNA expression vectors were co-transfected with luciferase reporter construct in HEK293T cells. Luciferase assay was performed 48 h post-transfection. Relative luciferase activity was normalized against *Renilla* luciferase activity. Relative -fold change was calculated relative to relative luciferase activity of the EV control. *E*, the miR-124 target site in Ezh2 3′-UTR is highly conserved among vertebrates. Alignment of the predicted miR-124 binding site in Ezh2 3′UTR of different species is shown (*Mmu*, *Mus musculus*; *Hsa*, *Homo sapiens*; *Rno*, *Rattus norvegicus*; *Ocu*, *Oryctolagus cuniculus*; *Ptr*, *Pan troglodytes*; *Cfa*, *Canis familiaris*; *Oga*, *Otolemur garnettii*; *Mml*, *Macaca mulatta*; *Eca*, *Equus caballus*; *Bta*, *Bos taurus*). *F*, miR-124 target sites in Ezh2 3′-UTR was analyzed by luciferase reporter. Mutations of the miR-124 target site in Ezh2 3′-UTR specifically abolish miR-124-mediated down-regulation of the luciferase reporter. The seeding region of the miR-124 target site in Ezh2 3′-UTR was either mutated by deletion (*Del 3*′*UTR*) or substitution (*Sub 3*′*UTR*) or left intact (*WT 3*′*UTR*). The -fold change was calculated relative to the relative luciferase activity of the reporter with wild-type Ezh2 3′-UTR in the absence of additional miRNA expression (*EV*). All data shown in the *bar graph* are mean ± S.D. (*error bars*) of at least three independent experiments. The differences between groups were first determined by analysis of variance (*B* and *D*), and the significance of miRNA-mediated down-regulation compared with the control was determined by a two-tailed Student's *t* test with equal variance (*, *p* < 0.05; **, *p* < 0.01; ***, *p* < 0.001).

To examine the extent of miRNA-dependent regulation of Ezh2 expression, we co-expressed an EGFP-Ezh2 3′-UTR reporter construct with each of the 30 miRNAs (including miR-124) predicted to interact with Ezh2 3′-UTR by five miRNA target prediction algorithms (Target Scan, PicTar, miRBase, miRNA.org, and MicroInspector) or nervous system-specific miRNA miR-9 (41) lacking the predicted binding sites in the Ezh2 3′-UTR. FACS analysis showed that the miR-124-induced down-regulation of the EGFP-Ezh2 3′-UTR expression exceeded that of most other miRNAs and was comparable with the effects induced by miR-26a and miR-101 (Fig. 2, *A* and *B*), two miRNAs previously reported to regulate Ezh2 expression and contribute to tumorigenesis (42, 43). Moreover, overexpression of miR-124, as well as miR-26a and miR-101, caused a noticeable down-regulation of endogenous Ezh2 protein level in HEK293T cells (Fig. 2*C*). The miR-9 control had no effect on the EGFP-Ezh2 3′-UTR expression and the expression of endogenous Ezh2 protein, as expected. These findings were further confirmed by a secondary screen with a luciferase Ezh2 3′-UTR reporter co-transfected with miR-124 or several other high scoring miRNA candidates (Fig. 2*D*). Notably, disruption of the predicted evolutionarily conserved miR-124 target site in the Ezh2 3′-UTR (Fig. 2*E*) by substitution or deletion (32, 44) abolished the miR-124-mediated down-regulation effect (Fig. 2*F*). We concluded that miR-124 is among the most efficient miRNA regulators of Ezh2 expression.

##### miR-124 Down-regulates Expression of Ezh2 but Not Other PRC2 Components during Neuronal Differentiation

**FIGURE 3.**
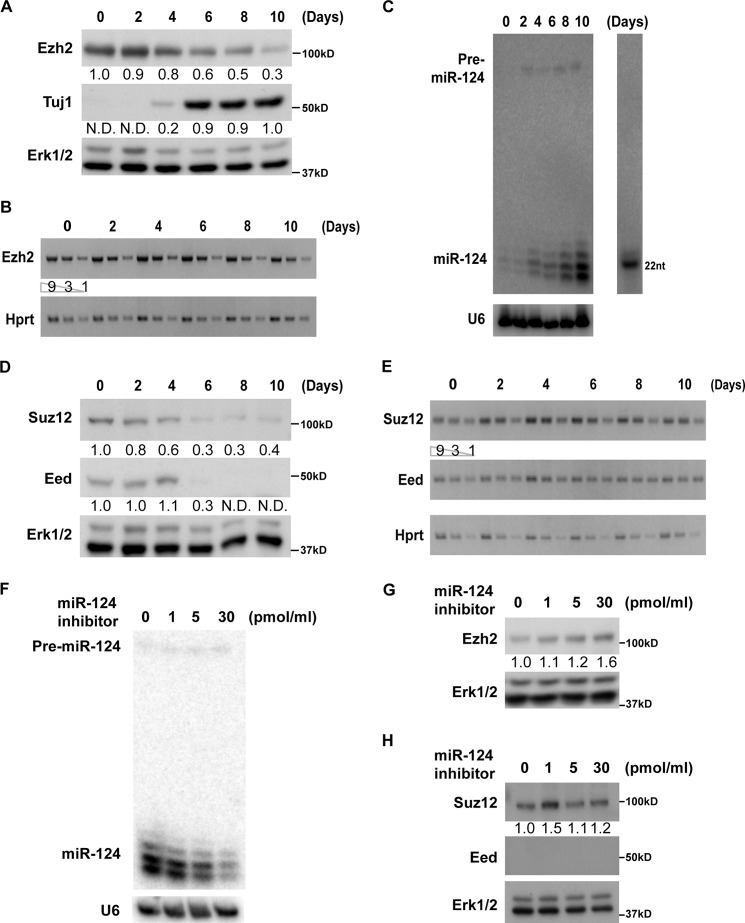
**miR-124 down-regulates Ezh2 expression during P19 neuronal differentiation.** *A–E*, inverse expression patterns of PRC2 members and miR-124 during P19 neuronal differentiation. Neuronal differentiation of P19 cells was induced by 1 μm all-*trans*-RA treatment for 4 days. Cell aggregates were dissociated into single cell suspensions at day 4 and recultured in neurobasal medium with N2 supplement. Cells were further cultured and collected at the indicated time points. *A*, Ezh2 and Tuj1 protein levels were analyzed by Western blot, and Erk1/2 served as a loading control. *B*, expression of *Ezh2* mRNA level was determined by semiquantitative RT-PCR. *Hprt* served as a loading control. *C*, increased miR-124 expression level during P19 neuronal differentiation. miR-124 expression levels were determined by Northern blot, and U6 RNA served as a loading control. A representative figure of two independent experiments is shown. This observation is shown in an earlier report (41). *D*, Suz12, Eed, and Tuj1 protein levels were analyzed as described above. *E*, expression of *Suz12* and *Eed* mRNA levels was analyzed by semiquantitative RT-PCR. HPRT served as a loading control. *F–H*, miR-124 inhibitor up-regulates endogenous Ezh2. miR-124 inhibitor was transfected into differentiating P19 cells at day 6 after the start of RA treatment. The levels of miR-124 and PRC2 members were analyzed at day 10. *F*, down-regulation of miR-124 expression level by inhibitor was confirmed by Northern blot analysis. A representative figure of two independent experiments is shown. The miR-124-specific inhibitor has been tested and published previously (11). *G*, Ezh2 protein expression was up-regulated upon miR-124 inhibitor treatment. *H*, Suz12 protein level was not altered upon miR-124 inhibitor treatment, whereas Eed protein level was below the detection limit of the Western blot assay. Ezh2, Suz12, Eed, and Tuj1 protein expression levels were normalized to Erk1/2. Their -fold changes were calculated relative to the protein levels at day 0 (*Ezh2* and *Suz12*), day 10 (*Tuj1*), or in non-transfected controls (*G* and *H*) and indicated *below* their respective blots. *N.D.*, not detectable. All data shown, unless otherwise stated, are representative of at least three independent experiments.

To examine whether physiological levels of miR-124 could regulate Ezh2 expression during neuronal differentiation, we took advantage of the retinoic acid (RA)-induced P19 embryonic carcinoma *in vitro* differentiation model (45, 46). We found that the Ezh2 protein level was noticeably down-regulated in P19 cells undergoing neuronal differentiation (Fig. 3*A*), whereas *Ezh2* mRNA levels remained virtually unchanged (Fig. 3*B*), thus indicating possible involvement of a post-transcriptional regulatory mechanism. Indeed, we found that miR-124 expression was inversely correlated with Ezh2 expression during P19 neuronal differentiation (Fig. 3*C*). Interestingly, mRNA and protein expression dynamics of two other PRC2 components, Suz12 and Eed, showed similar trends (Fig. 3, *D* and *E*).

Because, unlike *Ezh2*, the *Suz12* and *Eed* 3′-UTRs lacked putative miR-124 target sites, we hypothesized that miR-124 may directly down-regulate Ezh2 during neuronal differentiation, whereas Suz12 and Eed are probably regulated by distinct post-transcriptional mechanisms. To test this prediction, we disrupted the activity of endogenous miR-124 with a miR-124-specific locked nucleic acid antisense oligonucleotide in differentiating P19 cultures (47) (Fig. 3*F*). This treatment successfully restored Ezh2 but not Suz12 and Eed expression in a dose-dependent manner (Fig. 3, *G* and *H*). Although the amount of miR-124 was significantly reduced by treatment with a high dose inhibitor, the remaining miR-124 was still sufficient to partially suppress Ezh2 expression. It is therefore difficult to achieve more profound changes in Ezh2 protein levels through treatment with miR-124 inhibitor. These results, however, suggested that Ezh2 is the only PRC2 member directly regulated by miR-124.

##### miR-124-regulated Ezh2 Expression Is Critical for Efficient Neuronal Differentiation

**FIGURE 4.**
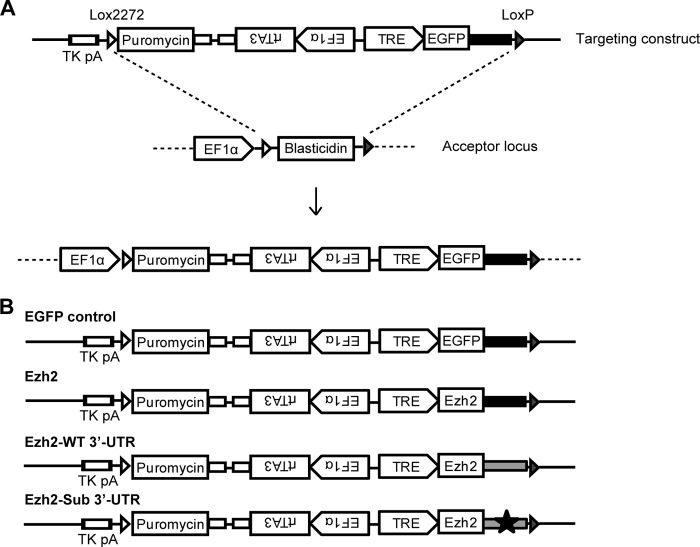
**Generation of doxycycline-inducible P19 cell lines expressing Ezh2.** *A*, targeting strategy for the generation of doxycycline (*Dox*)-inducible P19 cell lines expressing Ezh2. The diagram shows the targeting construct and the acceptor locus before and after Cre-recombinase-mediated recombination. The *empty* and *filled arrowheads* indicate the LoxP2272 and LoxP sequence, respectively. *B*, schematic presentation of various targeting constructs that were used to generate Dox-inducible P19 cell lines expressing Ezh2 with various 3′-UTRs. EGFP was used as a control for the inducible system. *Filled black boxes* denote artificial β-globin 3′-UTR (EGFP control and Ezh2). *Filled gray boxes* designate Ezh2 3′-UTR (*Ezh2 WT 3*′*UTR*). *Black star*, mutation in the miR-124 target site of wild-type 3′-UTR (*Ezh2 Sub 3*′*UTR*). Puromycin was used as a positive selection marker for the screening of Cre-recombinase-mediated recombination events in P19 HILO-RMCE acceptor cell line (33). *EF1*α, elongation factor-1α promoter; *TRE*, tetracycline response element; *rtTA3*, reverse tetracycline transactivator.

To address functional significance of miR-124-induced Ezh2 down-regulation during neuronal differentiation, we generated several transgenic P19 lines stably expressing doxycycline-inducible *Ezh2* mRNAs with various 3′-UTRs (Fig. 4, *A* and *B*). To avoid position effects caused by random integration, we utilized a site-specific transgene integration procedure developed earlier (33). This well characterized P19 HILO-RMCE acceptor cell line, where all constructs are targeting to the same locus, provides a superior experimental system for comparing the effects of various Ezh2 constructs on neuronal differentiation. An additional cell line expressing EGFP with Globin 3′-UTR (EGFP) was generated as a control.

**FIGURE 5.**
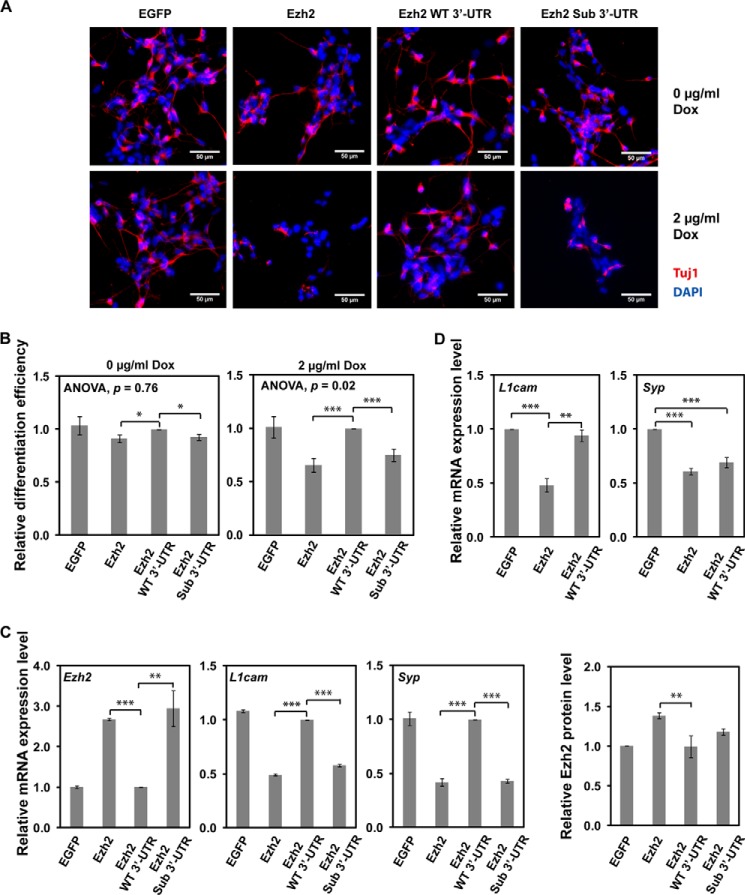
**miR-124-regulated Ezh2 expression is important for P19 neuronal differentiation.** Neuronal differentiation of various stable P19 cell lines was induced by RA treatment for 4 days and followed by the addition of Dox to promote the expression of Ezh2. Cells were analyzed at day 6.5. *A*, expression of miR-124 uncontrollable Ezh2 (*Ezh2* or *Ezh2 Sub 3*′*UTR*) inhibited neuronal differentiation. Neuronal population was defined by Tuj1 staining (*red*), and total cell number was determined by DAPI staining (*blue*). Representative images are shown. *Scale bar*, 50 μm. *B*, statistical analysis of neuronal differentiation. The efficiency of neuronal differentiation was calculated relative to that of Ezh2 WT 3′-UTR-expressing cells. The differences between groups were first determined by analysis of variance, and the significant difference between indicated pairs was determined by a two-tailed Student's *t* test with equal variance. Data shown are mean ± S.D. (*error bars*) of three independent experiments. *C*, RT-qPCR analysis for the expression of *Ezh2*, *L1cam*, and *Syp* in the indicated P19 stable cell lines. Data are normalized against *Hprt* expression and corresponding cell lines without Dox treatment. Relative mRNA expression levels of the indicated genes were calculated as -fold change compared with the gene expression in cells expressing Ezh2 WT 3′-UTR. A representative result with experimental triplicates from three independent experiments is shown. Data shown are mean ± S.D. of triplicates. Ezh2 protein levels (*far right*) in cells expressing various Ezh2 constructs were normalized to Tubulin, and -fold change was calculated relative to the Ezh2 protein level in cells expressing EGFP control. Data shown are mean ± S.D. of quantifications from three Western blots. *D*, RT-qPCR analysis for the expression of *L1cam* and *Syp* in cultured embryonic mouse neural stem cells. Data are normalized against *Hprt* expression. Relative mRNA expression levels of the indicated genes were calculated as -fold change compared with the gene expression in EGFP control cells. A representative result with experimental triplicates from three independent experiments is shown. Data shown are mean ± S.E. (*error bars*). Significant differences between indicated pairs in all *panels* were determined by two-tailed Student's *t* test with equal variance (*, *p* < 0.05; **, *p* < 0.01; ***, *p* < 0.001).

Using the expression of neuronal Tubulin β_III_ (Tuj1 immunofluorescence; Fig. 5, *A* and *B*) and expression of neuron-specific mRNAs, *L1cam* and *Syp*, as a readout (Fig. 5*C*), we found that expression of the *Ezh2* transgene (Ezh2) lacking its natural 3′-UTR reduced the efficiency of P19 neuronal differentiation. Conversely, the *Ezh2* transgene with the Ezh2 3′-UTR containing the miR-124 target site (Ezh2 WT 3′-UTR) failed to up-regulate Ezh2 protein level and cause this inhibitory effect (Fig. 5, *B* and *C*). Moreover, the efficiency of P19 neuronal differentiation was significantly reduced by the Ezh2 Sub 3′-UTR transgene lacking the miR-124 target site in its Ezh2-derived 3′-UTR. Our RT-qPCR and immunoblot analyses confirmed that these biological effects were accompanied by corresponding changes in the Ezh2 expression levels (Fig. 5*C*). Similarly, transient expression of recombinant Ezh2 in mouse embryonic neural stem cells undergoing neuronal differentiation down-regulated neuronal markers *L1cam* and *Syp* (Fig. 5*D*). On the other hand, expression of Ezh2 with WT 3′-UTR lowered expression of these genes to a lesser extent, an effect that was especially obvious for *L1cam* (Fig. 5*D*). Thus, down-regulation of Ezh2 protein expression by miR-124 is critical for efficient neuronal differentiation.

##### Ezh2 Regulation by miR-124 Balances Neurogenesis Versus Astrogenesis

**FIGURE 6.**
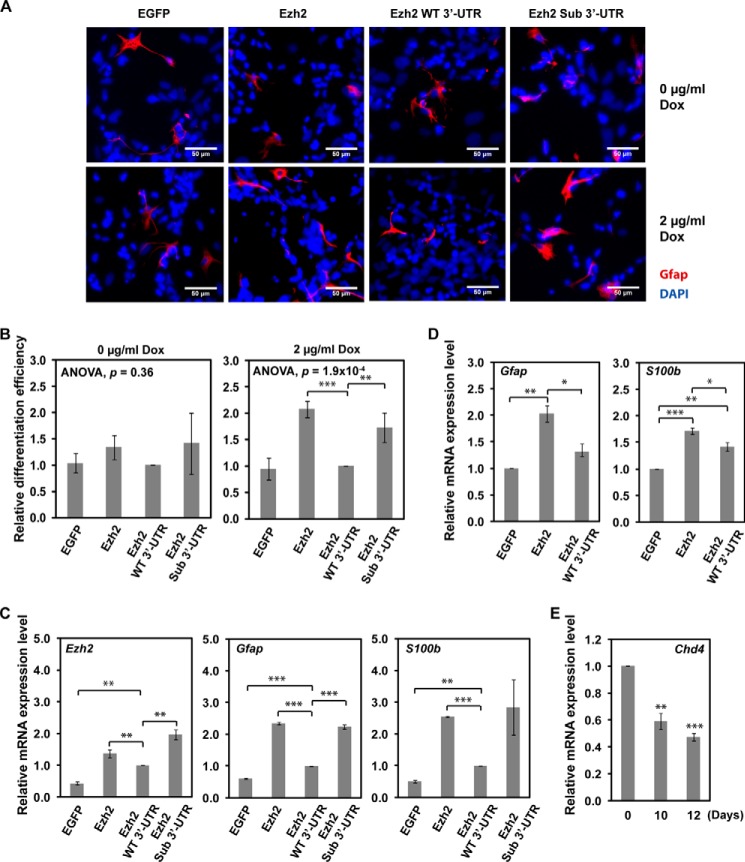
**Ezh2 overexpression promotes P19 astrocyte differentiation.** Neuronal differentiation of various stable P19 cell lines was induced as described in the legend to Fig. 5. Cells were analyzed at day 12. *A*, expression of miR-124 uncontrollable Ezh2 (*Ezh2* or *Ezh2 Sub 3*′*UTR*) promotes astrocyte differentiation. Astrocytes were defined by Gfap staining (*red*), and total cell number was determined by DAPI staining (*blue*). Representative images are shown. *Scale bar*, 50 μm. *B*, statistical analysis of astrocyte differentiation. The efficiency of astrocyte differentiation was calculated relative to that of Ezh2 WT 3′-UTR-expressing cells. The differences between groups were first determined by analysis of variance, and the significant difference between indicated pairs was determined by a two-tailed Student's *t* test with equal variance. Data shown are mean ± S.D. (*error bars*) of three independent experiments. *C*, RT-qPCR analysis for the expression of *Ezh2*, *Gfap*, and *S100b* in the indicated P19 stable cell lines. Data are normalized against *Hprt* expression and corresponding cell lines without Dox treatment. Relative mRNA expression levels of the indicated genes were calculated as -fold change compared with the gene expression in cells expressing Ezh2 WT 3′-UTR. A representative result from three independent experiments with experimental triplicates is shown. Data shown are mean ± S.D. of triplicates. *D*, RT-qPCR analysis for the expression of *Gfap* and *S100b* in cultured embryonic mouse neural stem cells. Data are normalized against *Hprt* expression. Relative mRNA expression levels of the indicated genes were calculated as -fold change compared with the gene expression in EGFP control cells. Data shown are mean ± S.E. (*error bars*) of three independent experiments. *E*, down-regulation of *Chd4* in differentiating P19 cells at the indicated time points was analyzed by RT-qPCR and normalized against *Hprt*. A representative result with experimental triplicates of three independent experiments is shown. Data shown are mean ± S.D. of triplicates. Significant differences between indicated pairs in all *panels* were determined by two-tailed Student's *t* test with equal variance (*, *p* < 0.05; **, *p* < 0.01; ***, *p* < 0.001).

miR-124 has been shown previously to promote neurogenesis and hinder gliogenesis using an *in vitro* differentiation model (48). Similarly, Ezh2-deficient neural progenitor cells are known to possess higher neurogenic and lower astrogenic potentials than their wild-type counterparts (49). To determine whether the miR-124/Ezh2 circuitry could control the choice between the two differentiation scenarios, we followed an established P19 astrogenesis protocol (50) and allowed transgenic P19 cells to differentiate for 12 days. We found that the efficiency of astrocyte differentiation was significantly enhanced in P19 cells expressing the miR-124-resistant Ezh2 Sub 3′-UTR transgene but not the miR-124-repressible Ezh2 WT 3′-UTR transgene (Fig. 6, *A* and *B*). The efficiency of astrocyte differentiation was determined by Gfap inmmunofluorescence staining and further verified by RT-qPCR for the expression of astrocyte-specific gene *S100b* (Fig. 6*C*). A similar astrogenesis-promoting effect was observed in cultured embryonic mouse neural stem cells expressing miR-124-resistant Ezh2 as well (Fig. 6*D*). Although Ezh2 is reported to prevent premature astrocyte differentiation in neurogenic phase by repressing astrocyte-specific genes in a Chd4-dependent manner (51), the *Chd4* expression level was down-regulated at this late stage of P19 culture (Fig. 6*E*). The elevated recombinant Ezh2 expression at this stage therefore did not suppress but rather promoted astrocyte generation. Our results suggest that miR-124-dependent regulation of Ezh2 expression might be critical for a balanced production of astrocytes and neurons.

## DISCUSSION

Neuron-enriched miRNA miR-124 provides a compelling example of a non-coding RNA modulating cellular gene expression at multiple levels (23). Importantly, this miRNA targets several master regulators of elaborated transcriptional and post-transcriptional programs, including transcription factor Sox9 (24), transcriptional co-repressor SCP1 (26), chromatin remodeling component BAF53A (27), and RNA-binding protein Ptbp1 (11). Here we expand this list by showing that in cells undergoing neural differentiation (P19 as well as embryonic mouse neural stem cells), miR-124 represses the expression of a critical epigenetic factor, lysine methyltransferase Ezh2. We provide evidence that Ezh2 down-regulation by miR-124 in this context promotes neuronal and counters astrocyte-specific differentiation route.

What could be a molecular mechanism underlying this effect? Ezh2 is known to limit neurogenic competence of neural progenitor cells and repress expression of several neurogenesis-promoting genes (30, 31, 49, 52), including master regulator for neuronal lineage commitment and differentiation *Ascl1*/*Mash1* (53, 54). Up-regulation of this gene is sufficient to promote neuronal differentiation (55, 56). Notably, *Ascl1* is one of the genes consistently derepressed by miR-124 in N2a neuroblastoma cells (Table 1), and our future studies will determine the functional significance of this effect.

**TABLE 1:** **miR-124-up-regulated Ezh2 target genes** Among miR-124-up-regulated genes (-fold change ≥1.5 and *p* < 0.001), 74 genes are Ezh2 target genes, and 52 of them are CNS-specific genes as defined in Ref. 39. FC, -fold change.

Gene symbol	Gene name	FC	CNS-specific
*Morc4*	Microrchidia 4	2.932	No
*Tpm1*	Tropomyosin 1, α	2.705	Yes
*Col8a2*	Collagen, type VIII, α2	2.494	No
*Dusp8*	Dual specificity phosphatase 8	2.371	Yes
*Atf3*	Activating transcription factor 3	2.244	Yes
*Prlr*	Prolactin receptor	2.128	Yes
*Mt1*	Metallothionein 1	2.102	Yes
*Smox*	Spermine oxidase	2.096	Yes
*Klhl22*	Kelch-like 22 (*Drosophila*)	2.070	Yes
*Plxna2*	Plexin A2	2.054	Yes
*Fbn2*	Fibrillin 2	1.990	Yes
*Sema7a*	Sema domain, immunoglobulin domain (Ig), and GPI membrane anchor, (semaphorin) 7A	1.989	Yes
*Igfbp6*	Insulin-like growth factor-binding protein 6	1.946	Yes
*Igfbp5*	Insulin-like growth factor binding protein 5	1.925	Yes
*Atp1a3*	ATPase, Na^+^/K^+^ transporting, α3 polypeptide	1.892	Yes
*Chrm3*	Cholinergic receptor, muscarinic 3, cardiac	1.862	No
*Tgfb3*	Transforming growth factor, β3	1.852	Yes
*Adora2b*	Adenosine A2b receptor	1.841	Yes
*Ap3m2*	Adaptor-related protein complex 3, μ2 subunit	1.839	Yes
*Shroom3*	Shroom family member 3	1.830	Yes
*Oxt*	Oxytocin	1.816	Yes
*Gnao1*	Guanine nucleotide-binding protein, αO	1.780	Yes
*Kcnk9*	Potassium channel, subfamily K, member 9	1.777	No
*Crhbp*	Corticotropin-releasing hormone-binding protein	1.768	Yes
*Slc35f1*	Solute carrier family 35, member F1	1.749	Yes
*Faah*	Fatty acid amide hydrolase	1.746	Yes
*Nphs2*	Nephrosis 2 homolog, podocin (human)	1.743	No
*Pax7*	Paired box gene 7	1.729	No
*Sypl2*	Synaptophysin-like 2	1.718	Yes
*Gpc5*	Glypican 5	1.714	Yes
*Kcne3*	Potassium voltage-gated channel, Isk-related subfamily, gene 3	1.707	No
*Sp7*	Sp7 transcription factor 7	1.705	Yes
*Epha5*	Eph receptor A5	1.703	Yes
*Gpc4*	Glypican 4	1.701	Yes
*Itpka*	Inositol 1,4,5-trisphosphate 3-kinase A	1.691	Yes
*Gprc5c*	G protein-coupled receptor, family C, group 5, member C	1.682	No
*Irx6*	Iroquois-related homeobox 6 (*Drosophila*)	1.680	No
*Hhat*	Hedgehog acyltransferase	1.678	No
*Gpr45*	G protein-coupled receptor 45	1.678	Yes
*Celsr2*	Cadherin, EGF LAG seven-pass G-type receptor 2 (flamingo homolog, *Drosophila*)	1.664	Yes
*Kirrel3*	Kin of IRRE like 3 (*Drosophila*)	1.662	Yes
*Cyp46a1*	Cytochrome P450, family 46, subfamily a, polypeptide 1	1.661	Yes
*Hoxc12*	Homeobox C12	1.656	No
*Tcfap2b*	Transcription factor AP-2β	1.640	No
*Nkx1-2*	NK1 transcription factor-related, locus 2 (*Drosophila*)	1.638	No
*Nol3*	Nucleolar protein 3 (apoptosis repressor with CARD domain)	1.632	Yes
*Rab15*	RAB15, member RAS oncogene family	1.630	Yes
*Nuak2*	NUAK family, SNF1-like kinase, 2	1.629	No
*Tmem28*	Transmembrane protein 28	1.628	Yes
*Hmx1*	H6 homeobox 1	1.625	No
*Spryd3*	SPRY domain-containing 3	1.624	Yes
*Btbd11*	BTB (POZ) domain containing 11	1.613	Yes
*Adamtsl5*	ADAMTS-like 5	1.610	No
*C1qtnf4*	C1q and tumor necrosis factor related protein 4	1.607	Yes
*Cacna2d2*	Calcium channel, voltage-dependent, α2/δ subunit 2	1.603	Yes
*Dpp10*	Dipeptidylpeptidase 10	1.594	Yes
*Zmiz1*	Zinc finger, MIZ-type-containing 1	1.591	Yes
*Hoxa9*	Homeobox A9	1.589	No
*Calb1*	Calbindin 1	1.585	Yes
*Pstpip2*	Proline-serine-threonine phosphatase-interacting protein 2	1.583	Yes
*Plagl1*	Pleiomorphic adenoma gene-like 1	1.581	Yes
*Nkx2-6*	NK2 transcription factor related, locus 6 (*Drosophila*)	1.579	No
*Trim54*	Tripartite motif-containing 54	1.562	No
*Gpr6*	G protein-coupled receptor 6	1.552	Yes
*Apln*	Apelin	1.547	Yes
*Snx22*	Sorting nexin 22	1.534	Yes
*Alx4*	Aristaless-like homeobox 4	1.534	No
*Nxph4*	Neurexophilin 4	1.526	No
*En2*	Engrailed 2	1.522	Yes
*Tmem25*	Transmembrane protein 25	1.520	Yes
*Tubb2b*	Tubulin, β2B	1.513	Yes
*Ybx2*	Y box protein 2	1.506	No
*Il12rb1*	Interleukin 12 receptor, β1	1.505	Yes
*Ascl1*	Achaete-scute complex homolog 1 (*Drosophila*)	1.502	Yes

Our analysis also revealed that more than 1800 Ezh2 target genes are not up-regulated in miR-124-overexpressing cells. Non-CNS-specific genes (634 genes) are probably associated with silent chromatin in neuronal progenitors and therefore could not be up-regulated simply by miR-124-mediated Ezh2 down-regulation. A small fraction of Ezh2 target genes with predicted miR-124 target sites could potentially be down-regulated by miR-124 (81 genes; 7 non-CNS and 74 CNS-specific), but only 12 CNS-specific genes were down-regulated in miR-124-overexpressing cells, which is not statistically enriched. The remaining 1242 CNS-specific genes may require additional CNS-specific activators that are not expressed just 24 h after miR-124 transfection. Although our current analysis already revealed a significant overlap between Ezh2 target genes and the miR-124 up-regulated gene list, more Ezh2 target genes could be up-regulated by persistent miR-124 overexpression in differentiating neurons.

Interestingly, examination of published genomic maps of the Ezh2-specific 3meH3K27 modifications suggests that promoter regions of all three mouse *miR-124* genes are associated with this repressive mark as well as Suz12, a component of the PRC2 complex, in ES cells (Table 2) (52). It is therefore possible that Ezh2 controls miR-124 levels in stem cells, synergizing with the repressive effect of REST (57). During the neurogenic phase, the H3K27-specific demethylase Jmjd3 is up-regulated, leading to derepression of neuron-specific genes, possibly including *miR-124* (58), that can now dampen Ezh2 expression. This hypothetical double-negative feedback between miR-124 and Ezh2/PRC2 would be similar to the previously reported relationship between miR-124 and SCP1/REST (26).

**TABLE 2:** **Enrichment of PRC2 at all three miR-124 loci** ChIP-seq data were selected from a previous publication (52), which shows the enrichment of Oct4, Sox2, Nanog, Tcf3, Suz12, and 3meH3K27 at different *miR-124* loci.

Loci	Positions of miR-124 promoter	Positions of Pre-miR-124	Suz12 binding sites	Other transcription factors or histone modification associated with miR-124 loci				
*mmu-mir-124-1*	chr14:63540450–63546275	chr14:63544772–63544848 (+)	chr14:63540776–63544525	Oct4		Nanog		3meH3K27
*mmu-mir-124-2*	chr3:17986635–17986835	chr3:17987829–17987903 (+)	chr3:17985751–17988075	Oct4	Sox2	Nanog	Tcf3	3meH3K27
*mmu-mir-124-3*	chr2:180819000–180825000	chr2:180823445–180823520 (+)	chr2:180820551–180823275	Oct4				3meH3K27

Although further work will be needed to address the miR-124-Ezh2/PRC2 cross-regulation model, the results of our preliminary studies are consistent with this possibility. Indeed, induction of Ezh2 expression following RA treatment led to dramatic down-regulation of miR-124 expression in differentiating P19 cultures (data not shown). Thus, it is possible that the stimulatory effect of miR-124-resistant Ezh2 on astrocyte generation observed in our study might be caused by Ezh2-mediated down-regulation of miR-124 expression. Underscoring the biological relevance of these regulatory events, miR-124 is naturally expressed in neurons but not in astrocytes (15), and it is predicted to directly down-regulate a number of astrocyte-enriched genes (22, 39).

In addition to its role in balancing neurogenesis *versus* astrogenesis, the miR-124/Ezh2 circuitry may function in other biological scenarios. For example, it has been recently proposed to control aggressiveness of hepatocellular carcinoma (32). Another recent study showed that miR-124 may prevent the activation of microglia, immune cells residing in the central nervous system (16). Interestingly, Ezh2 is known to be up-regulated in activated lymphocytes and play an essential role in this process (59, 60),^3^ and it would be interesting to examine the role of Ezh2 in the context of microglia activation, which contributes to pathogen clearance in health or the progression of neurodegenerative and neoplastic diseases (61).

Although we show here that miR-124 represents one of the most potent miRNA regulators of Ezh2 expression, our data are also consistent with the possibility of combinatorial regulation by miRNA. Other than miR-124, miR-26a, and miR-101, six additional miRNAs consistently down-regulated the expression of Ezh2 3′-UTR reporter genes (Fig. 2*D*). Of these, only miR-20a, miR-26a, and miR-124 are known to be up-regulated in differentiating P19 cells (62), which predicts possible synergistic effects of these three miRNAs on Ezh2 abundance. However, miR-124 is likely to be the major regulator of Ezh2 expression in differentiating neurons, because it is the most abundant miRNA in the brain (12) and is also highly up-regulated in differentiating P19 cells (20 times for miR-124 *versus* 2 times for miR-20 and miR-26a) (62). The binding sites of these miRNAs are not overlapping. Other miRNAs identified in our study do not appear to be relevant for differentiating neurons.

In conclusion, our study suggests that miRNA control of an important epigenetic regulator can be used as a regulatory paradigm for modulating the choice between alternative differentiation scenarios.

## Footnotes

3 W. H. Neo and I.-H. Su, unpublished data.

## Footnotes

2 The abbreviations used are:

miRNA: microRNA
NS: nervous system
H3K27: histone H3 Lys-27
3meH3K27: H3K27 trimethylation
N2a: Neuro2a
NSC: neural stem cell
RT-qPCR: quantitative RT-PCR
qPCR: quantitative PCR
RA: retinoic acid
EGFP: enhanced GFP.
